# Pilot study protocol of a randomized controlled trial for the potential effects of creatine monohydrate on persistent post-concussive symptoms

**DOI:** 10.3389/fneur.2023.1209548

**Published:** 2023-07-05

**Authors:** Ronni Lykke Bødker, Michael Marcussen

**Affiliations:** ^1^Commotio Denmark, Køge, Denmark; ^2^Department of Public Health, University of Southern, Odense, Denmark; ^3^Research unit of Psychiatry, Region Zealand, Slagelse, Denmark

**Keywords:** concussion, creatine monohydrate, persistent post-concussive symptoms, mild traumatic brain injury, nutrition, Rivermead post-concussion symptoms questionnaire

## Abstract

**Background:**

Mild traumatic brain injury or concussion is a global public concern, with an estimated annual incidence between 48 million and 96 million worldwide. It is a socioeconomical problem, and almost one-third of individuals with concussion suffer from severe persistent post-concussive symptoms (PPCS), with an increased risk of unemployment or terminating their studies. To date, no single treatment is available with guaranteed success. Creatine monohydrate (CrM) has shown potential as a treatment for post-concussive symptoms, having a positive impact on cognitive function, chronic fatigue, depression, and anxiety. The aim of this study is to examine the effect of CrM on PPCS assessed using the Rivermead Post-Concussion Symptoms Questionnaire (RPQ).

**Methods:**

The study is designed as a double-blinded randomised controlled trial. Study participants are found through neurological outpatient clinics in Denmark or through social media. They will be between 25 and 35 years of age, will have suffered from PPCS for 6–12 months prior to inclusion, and will have no comorbidities. The participants will be randomly allocated to either an intervention group (INT), placebo group (PLA), or control group (CG). Baseline data will be collected immediately after inclusion, and the study period will be 7 weeks. Follow-up data will be collected 1 week after the end of the study period. The primary outcome of the study is changes in RPQ score. Changes in weight and training status will be adjusted for as potential confounders.

**Ethics and dissemination:**

This protocol is approved by the National Committee on Health Research (97508) and by the Danish Data Protection Agency 11.651. The investigators intend to submit their study findings for publication in peer-reviewed journals and disseminate the findings via presentation at academic meetings/conferences.

**Clinical Trial registration**: NCT05562232, registered September 30, 2022.

## Introduction

Mild traumatic brain injury (mTBI), which is used interchangeably with ‘concussion’ in the literature (1), is a significant public concern. It is estimated that between 0.6 and 1.2% of the general population will suffer an mTBI each year (2, 3), which represents 48–96 million people on a global scale. Of these patients, an estimated 10–30% will suffer from persistent post-concussive symptoms (PPCS) (4, 5). These symptoms typically include headache, poor concentration, memory problems, fatigue, sleep difficulties, dizziness, irritability, and feeling nervous or anxious (4, 6). PPCS is not only a health problem, but also a socioeconomical problem (7). Data from Fallesen et al. (7) revealed that the salary of a concussion patient in Denmark 5 years after their concussion decreased 4.2% and was associated with an increased risk of becoming unemployed. To date, no single treatment option has been made available with guaranteed success; therefore, nutritional supplements represent an alternative.

The nutritional supplement creatine monohydrate (CrM) is one of the most popular ergogenic aids on the market among professional and amateur athletes (8). It is mostly used in the development of muscle mass, as creatine is primarily found in skeletal muscle (9). Roughly 5% of the body’s creatine is distributed in the brain and testicles (9). As mTBI alters the metabolism of the brain (10–12), creatine supplementation might be beneficial for patients with PPCS (13). Although studies have shown little evidence for alterations in brain creatine in the acute phase (13). This is further supported by a study suggesting that creatine supplementation may reduce the severity of mild concussion in animal models (14).

Furthermore, because the enzyme creatine kinase (CK), which is involved in the ATP energy system, also has a brain specific isoform (BB-CK) (15), creatine may be a relevant component the energy system of the central nervous system (16). In addition, several studies suggests that creatine supplementation increases cellular energy availability (17). Creatine has been reported to increase brain phosphocreatine content by as much as 15%, which in turn improves the metabolic processes of the brain (14, 18–21).

CrM has been described to be a potent anti-inflammatory molecule (22, 23). It has been shown to reduce cytotoxic effects in cells that have undergone oxidative injury without affecting antioxidant enzyme activity (23), and it has also been shown to inhibit the reactive oxygen species-induced formation of mitochondrial permeability transition pores in the liver mitochondria of mice (22). Moreover, concussion seems to increase inflammation in the brain (24), and this inflammation has been hypothesised to correlate with the symptomatology and duration of the concussion (25). Even though research in the area of recovery is scarce, neuroinflammation seems to play a vital role in the pathophysiology of concussions (24). This warrants hopes of a decrease in post-concussive symptoms following CrM supplementation.

Other studies have indicated that CrM improves cognitive function, including fatigue (26), working memory (27, 28), and mood state (17), which are all symptoms associated with PPCS (4). Additional evidence suggests that creatine supplementation can help with chronic fatigue (17), depression (29), and anxiety (17). Therefore, CrM may be helpful in the treatment of PPCS.

Currently, there is no viable treatment option for PPCS patients. However, if CrM supplementation shows positive results, patients suffering from PPCS would be able to reduce their symptoms in a relatively easy and low-cost way.

*The aim* of this study is to investigate whether CrM as a supplement reduces the number and severity of symptoms in patients with PPCS through self-reported post-concussion symptoms questionnaires.

## Methods and analysis

### Study design

The pilot study will be performed as a randomised controlled trial in accordance with the SPIRIT guidelines (30). The study will include a convenience sample of 45 patients. The patients will be randomly allocated to either a control group (CG), placebo group (PLA), or intervention group (INT), with 15 patients in each group. As there has been too few randomised controlled trials (RCT) to establish a sample size through power calculations, this pilot study will be used to establish a sample size for a future RCT.

The study will be double blinded, that is, the participants, staff and investigators, will be unaware of treatment group the patients are allocated to. Furthermore, the process of randomisation will consist of a nutritionist not otherwise associated with the study who will randomly allocate each participant to a group until the desired sample size is reached. The nutritionist in charge of this step will also distribute either the placebo or the CrM to the participants in each of these groups.

CG will not receive any treatment other than usual treatment. PLA and INT will both receive a powder that must be ingested. PLA will receive a powder similar in appearance to CrM, but with no apparent nutritional value. INT will receive CrM.

### Procedure

As both PLA and INT are the interventions, they will follow the same protocol for ingestion: 5 g per day for 7 weeks. All 5 g will be ingested at once (9). This protocol has been chosen instead of the more common 0.3 g/day for the first week ingested five times during the day (9). In other studies on CrM and the brain, 5 g per day was also the chosen strategy (31, 32). However, studies on CrM in the muscles indicate that both protocols illicit the same response at 28 days (9), and a loading phase is not required (33). Furthermore, some studies have used this protocol with creatine supplementation and reported increased cognitive function (27, 34). Our reason for choosing 5 g/day over 7 weeks was to increase compliance and decrease the risk of discomfort with high CrM intake. An intake five times a day for the first week is demanding and would likely result in some participants quitting the study.

At the mid-phase of the study, all baseline measurements will be repeated. The length of the intervention is 7 weeks. After the 7 weeks, all baseline measurements will be repeated (see Table 1). One week after last ingestion, the measurements will be performed one last time. At the week 8 appointment, every participant will be asked whether they thought they had received a placebo or CrM to determine how large an effect the placebo will have on the results.

**Table 1:** Study timeline.

	Pre (−1 week)			Intervention (7 weeks)			Post (+1 week)		
	CG	PLA	INT	CG	PLA	INT	CG	PLA	INT
Declaration of consent	X	X	X						
Height (m)	X	X	X				X	X	X
Weight (kg)	X	X	X	X	X	X	X	X	X
Concussion history	X	X	X						
Training status (hours/week)	X	X	X				X	X	X
RPQ (35, 36)	X	X	X	X	X	X	X	X	X
CrM						X			
Placebo powder					X				
Usual treatment				X	X	X			

CG will receive standard care. However, to our knowledge there is no commonly accepted description of standard care for PPCS in the literature. In general, these participants will be advised to continue with daily regular routines during the 7 weeks.

### Study timeline

Table 1.

### Participants and recruitment

The study population will be patients who have been experiencing PPCS for 6–12 months at start of participation. Participants will be recruited through social media and 14 neurological outpatient clinics in Denmark. At the start of the study, participants will be between 25 and 35 years of age. This will make the population homogenous regarding age, and we will avoid the physical and cognitive challenges associated with early childhood and adolescents (37–39), the degeneration in physical capacity that begins at approximately 35 years of age (39), and the cognitive decline that begins around the same time (40).

The anthropometrics of the participants will consist of age (years), gender (male/female), height (cm), body mass (kg), period with PPCS (months), and training status (hours/week).

### Inclusion criteria

- Diagnosis of concussion/mild traumatic brain injury (1) from either the emergency care unit or general practitioner, with symptoms for a minimum of 6 months and a maximum of 12 months before the start of the inclusion.
- Age between 25 and 35 years.

### Exclusion criteria

- Elite athletes and people who are normally physically active for more than 10 h a week on average.
- Participation in other interventions/treatments beyond this study.
- Pregnancy.
- Moderate or severe traumatic brain injury.
- Current intake of CrM.

### Outcomes

- Primary

o PPCS will be measured using the Rivermead Post-Concussion Symptoms Questionnaire (RPQ). The RPQ is a questionnaire intended to quantify symptoms related to PPCS; it measures 16 commonly experienced symptoms following concussion. It is a comparison of pre-morbid levels with levels within the last 24 h (35). The patients will score each of the 16 symptoms from 0 (not experienced at all) to 4 (a severe problem). When all 16 symptoms are scored, the overall score is calculated by adding the scores together. A higher score is associated with a greater expected severity of PPCS (41). The first three questions concern (RPQ-3) the typical acute symptoms, while questions 4–16 (RPQ-13) concern the later symptoms manifesting after days or weeks (41).

- Secondary

o TBI/concussion history will be assessed using the Ohio State tool, a self-report questionnaire regarding lifetime history of head and neck injuries.
o Furthermore, we will monitor adverse events in line with general guidelines, even though we have found no evidence that CrM should have any adverse effects in the dose given (9).

### Potential confunders

- Body weight will be measured at baseline, halfway through the intervention, and 1 week after the end of the intervention. These measurements are performed to ensure that changes in body weight do not affect the results, as changes in body weight are common with CrM supplementation.
- Physical activity will be self-reported in hours/week throughout the study period, as this can be a relevant factor to measure.

In the pilot study we will not assess the risk factors, but in the future RCT study, we will assess generally accepted risk factors.

### Statistics

Scales will be assessed for internal reliability, and Cronbach’s alpha will be estimated. We will apply unpaired t-tests to assess mean score differences at baseline and chi-square tests for sex distribution. Differences over time will be explored using paired sample t-tests. Applying Cohen’s d, effect sizes will be derived by calculating mean differences and standard deviations. To assess differences in outcomes between groups, we will employ linear mixed regression. All statistical analyses will be performed using SPSS for Windows (IBM Corp., 2018., Version 25.0., Armonk, NY).

## Discussion

This study will be the first, to our knowledge, to investigate the effect of CrM on PPCS. We hypothesise that CrM supplementation will reduce the number and severity of PPCS measured though the self-reported post-concussion symptoms questionnaire called RPQ.

The short study period will prohibit conclusions on long-term impact. Furthermore, this study will not elucidate whether CrM is effective in the acute phase of a mild brain injury, such as concussion. This pilot study will therefore, in addition, serve as a methodological stepstone before investigating CrM as a possible treatment in larger populations.

If the large scaled RCT can elucidate that CrM is effective in the treatment of patients with PPCS, this could have a significant impact on the treatment courses offered by the healthcare system and followingly on the patients’ well-being.

## Dissemination

The investigators intend to submit their study findings for publication in peer-reviewed journals and to disseminate the findings via presentation at academic meetings/conferences.

## Ethics statement

This protocol was approved by the National Committee on Health Research Ethics (97508) and by the Danish Data Protection Agency 11.651. The study is registered at clinicaltrials.gov with the identifier NCT05562232.

The study will follow the ethical principles for medical research involving human subjects of the Declaration of Helsinki, adopted by the 18th General Assembly of the World Medical Association (42), which were last revised at the Association’s 64th General Assembly in Fortaleza, Brazil, in October 2013.

Before participation, the patients will be informed of the project and its purpose, both verbally and in writing. Response to the questionnaires constitute voluntary consent to participation; this will be applied for both baseline and follow-up. The participants will be required to sign a declaration of consent to ensure that they are fully aware of what they agree to participate in, the associated risks, and what they can gain from the study.

Participants will be free to withdraw from the study at any time without giving an explanation; if they choose to do so, their data will not be part of the final analysis. Data will be entered into the SurveyXact Online Clinical Trial Management System. Participants will receive a registration number as soon as they are deemed eligible for the study to ensure their anonymity. All personal identifiers will be removed or disguised during analysis to preclude personal identification.

There are no expected risks or harmful side effects from the study, as there no reported risks or harmful side effects of CrM supplementation, even with over 1 year of use (43). Furthermore, CrM supplementation is not expected to cause any discomfort for the participants.

## Author contributions

RB: conceptualisation, data curation, formal analysis, investigation, methodology, validation, writing—original draft, writing—review and editing. MM: conceptualisation, data curation, formal analysis, methodology, validation, writing—review and editing. All authors contributed to the article and approved the submitted version.

## Conflict of interest

The authors declare that the research was conducted in the absence of any commercial or financial relationships that could be construed as a potential conflict of interest.

## Publisher’s note

All claims expressed in this article are solely those of the authors and do not necessarily represent those of their affiliated organizations, or those of the publisher, the editors and the reviewers. Any product that may be evaluated in this article, or claim that may be made by its manufacturer, is not guaranteed or endorsed by the publisher.
